# Gamma Power Is Phase-Locked to Posterior Alpha Activity

**DOI:** 10.1371/journal.pone.0003990

**Published:** 2008-12-22

**Authors:** Daria Osipova, Dora Hermes, Ole Jensen

**Affiliations:** 1 Donders Institute for Brain, Cognition and Behaviour, Radboud University Nijmegen, Nijmegen, The Netherlands; 2 Section Brain Function and Plasticity, Department of Neurology and Neurosurgery, Rudolf Magnus Institute of Neuroscience, University Medical Center Utrecht, Utrecht, The Netherlands; University of Minnesota, United States of America

## Abstract

Neuronal oscillations in various frequency bands have been reported in numerous studies in both humans and animals. While it is obvious that these oscillations play an important role in cognitive processing, it remains unclear how oscillations in various frequency bands interact. In this study we have investigated phase to power locking in MEG activity of healthy human subjects at rest with their eyes closed. To examine cross-frequency coupling, we have computed coherence between the time course of the power in a given frequency band and the signal itself within every channel. The time-course of the power was calculated using a sliding tapered time window followed by a Fourier transform. Our findings show that high-frequency gamma power (30–70 Hz) is phase-locked to alpha oscillations (8–13 Hz) in the ongoing MEG signals. The topography of the coupling was similar to the topography of the alpha power and was strongest over occipital areas. Interestingly, gamma activity per se was not evident in the power spectra and only became detectable when studied in relation to the alpha phase. Intracranial data from an epileptic subject confirmed these findings albeit there was slowing in both the alpha and gamma band. A tentative explanation for this phenomenon is that the visual system is inhibited during most of the alpha cycle whereas a burst of gamma activity at a specific alpha phase (e.g. at troughs) reflects a window of excitability.

## Introduction

Neuronal oscillations in different frequency bands have been reported in multiple studies in both humans and animals. These oscillations are produced by large ensembles of neurons oscillating in synchrony and are considered to be important for neuronal computation responsible for e.g. perception, memory, and attention [1]–[4]. Much less is known about interactions between various frequency bands during specific cognitive tasks or simply at rest. This interaction can be carried out in several ways [5]: by amplitude correlations [6]–[8], phase synchronization (n∶m coupling) [8], or phase to power locking [7], [9]–[12]. Also bicoherence, that has been applied to animal [13] and human data [14], revealed cross-frequency interactions between theta and gamma activity.

Phase to power coupling is particularly interesting since it could be generated by a slower rhythm (e.g. theta or alpha) modulating the excitability of a network producing high frequency oscillations. In animals, phase to power interactions have been reported in the theta (5–8 Hz) and gamma (30–70 Hz) bands. This phenomenon has been identified in the hippocampus of anesthetized and awake rats [15], [16]. In humans, similar coupling has been reported with intracranial recordings in the medial temporal lobe during successful vs. unsuccessful memory performance [9], [12]. The theta phase to gamma power modulation was task dependent in various cognitive paradigms. Yet, a study applying intracranial electrodes in human neocortical areas reported that delta oscillations (0–3.5 Hz) oscillations modulated gamma power in distributed brain areas [7].

Spontaneous brain activity recorded from humans during rest is dominated by strong oscillations in the alpha band (8–13 Hz) [17]. These oscillations are produced in posterior brain regions in which gamma activity also has been reported [18]–[20]. In order to examine the interaction between cell assemblies producing the two main rhythms in the human visual system, we have investigated cross-frequency coupling between the phase of the alpha activity and the gamma power in the human magnetoencephalogram (MEG).

## Results

**Figure 1:**
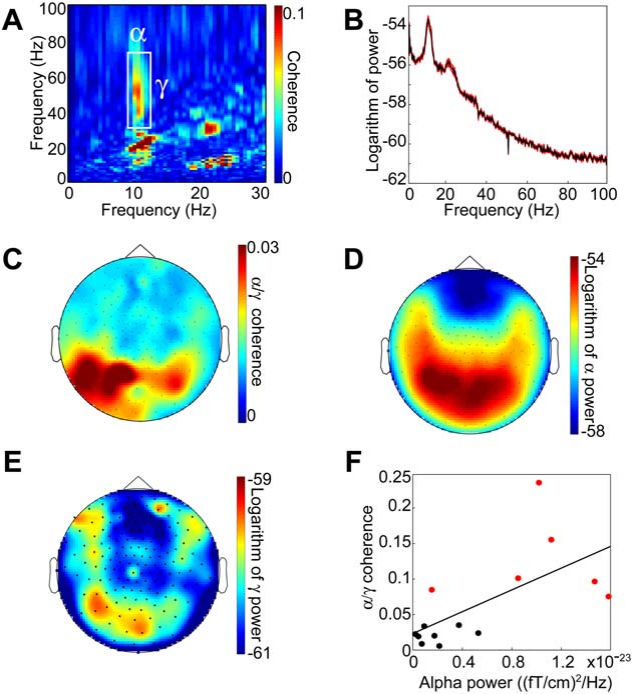
Coupling between alpha and gamma activity. A. Cross-frequency interaction in ongoing human MEG signals during eyes closed. The highlighted area indicates increased coherence between alpha activity (along the x-axis) and the power of the gamma activity (along the y-axis). B. Grand-average (black line) and standard deviation (red line) of log-transformed power. While a strong peak could be observed in the alpha band there was no detectable peak in the gamma band. C. Topography of cross-frequency coupling (the highlighted area in A). D. Topography of the log–transformed alpha power (9–11 Hz). E. Topography of the log–transformed gamma power (30–70 Hz). A to E are calculated from an average of 6 subjects. F. Correlation over 14 subjects between alpha power and the cross-frequency coupling in the gamma band. Mainly subjects with higher alpha power had significant cross-frequency interactions (shown in red).

The cross-frequency measure was applied to the sensor data in each subject to investigate coherence between a low frequency signal and the time-course of the power at higher frequencies. Interaction was observed between alpha and gamma bands. The sensors with the strongest alpha-gamma coupling were identified subject by subject. The human gamma-band activity reported in different studies varies from 30 to 150 Hz (for a review, see [2]), possibly depending on a cognitive task, imaging method used and/or intersubject differences. Therefore, the boundaries of the gamma-band of a single subject may somewhat differ from those of the grand average. Cross-frequency coupling was significant in six subjects (four subjects: p<0.01; two subjects: p<0.05). Data from these subjects were subjected for further analysis. The cross-frequency representations were averaged showing coupling between the phase of alpha (8–12 Hz) and the power of gamma activity (30–70 Hz) (Fig. 1A). Note that the 10 to 20 Hz coupling is likely to be explained by the first harmonic of the alpha activity. The power spectra for those sensors were also averaged over subjects. Interestingly, while the alpha activity resulted in a strong 10 Hz peak, the gamma activity was not apparent as a peak in the spectrum (Fig. 1B). Then we extracted mean cross-frequency values for all sensors for a 8–12 Hz by 30–70 Hz tile (white rectangle in Fig. 1A). The corresponding topography averaged over sensors is shown in Fig. 1C. The posterior distribution resembled the topography of the ∼10 Hz alpha power (Fig. 1D). Next, we tested if the strength of the alpha power correlated with degree of cross-frequency coupling in all 14 subjects (Fig. 1F). This resulted in a significant correlation suggesting that strong alpha power (Spearman r = 0.68, p<0.01) is a prerequisite for the cross-frequency coupling. Although the differences in alpha-gamma coupling and alpha power between subjects cannot be attributed to the amount of data used in the analysis, it remains hard to dissociate whether inter-individual differences in the observed effect are due to differences in signal-to-noise or reflect an underlying physiological phenomenon. It is important to note, however, that the amount of data used in the present study resembles the amount of data used in a typical cognitive paradigm (100 trials of 1 s). Thus there is a realistic chance to detect cross-frequency coupling even in the absence of an extended recording.

**Figure 2:**
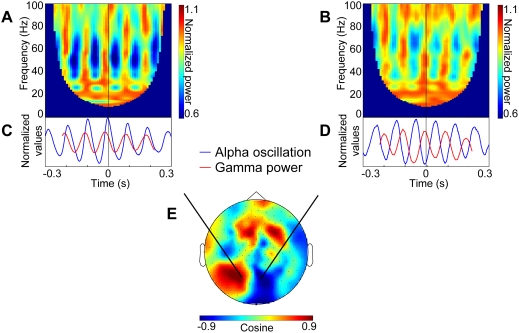
The phase difference between gamma power and alpha oscillation. A,B. Time-frequency representations of power for epochs phase aligned with respect to the alpha cycles in one subject. The strongest gamma band modulations were observed at 30–70 Hz. C,D. Time course of gamma power (30–70 Hz average) overlaid with respect to the averaged epochs of the phase-aligned alpha oscillation. Gamma power is in phase with alpha activity to the left (C) of the source and in anti-phase to the right (D). A,C are based on one channel on the left side of the source; B,D are based on one channel on the right side of the source. E. Topography of the phase difference (cosine of coherency; measured in radians) between the alpha activity and the gamma power.

To investigate the phase relationship between gamma power and the alpha signal we calculated time-frequency representations of power with respect to epochs aligned to the alpha phase (data from one subject shown in Fig. 2). Confirming the cross-frequency estimates, this revealed a strong modulation in gamma power (30–70 Hz) with respect to the alpha phase. Depending on either left or right hemisphere sensors gamma power was strongest at, respectively, alpha peaks or troughs (Fig. 2). This shows that the gamma power modulation is in phase or anti-phase with the alpha signal in respectively the left and right hemisphere. Further, based on the average of the four subjects whose data revealed this dipolar topography (data not shown), it seems more likely that gamma power coincides with peaks and troughs of alpha oscillation, rather than with its rising phase. However, we do not have the sufficient amount of data to make stronger claims about the phase lag. In the other two subjects the topography did not have a clear dipolar pattern.

Biophysically, the observed dipolar topography is best explained by alpha activity produced by a single source in the midline. This source will produce a dipolar field distribution (i.e. being in anti-phase in the axial gradiometers with respect to left and right hemisphere). Consistent with the notion that it is one single alpha source modulating the gamma power, the gamma power will be in phase in the left and right hemisphere.

The relationship between gamma power and alpha phase was also estimated from the angle of the coherency. Figure 2E shows the topography of the cosine of the coherency (i.e. the real value of coherency). This yielded a bipolar pattern over the occipital region.

**Figure 3:**
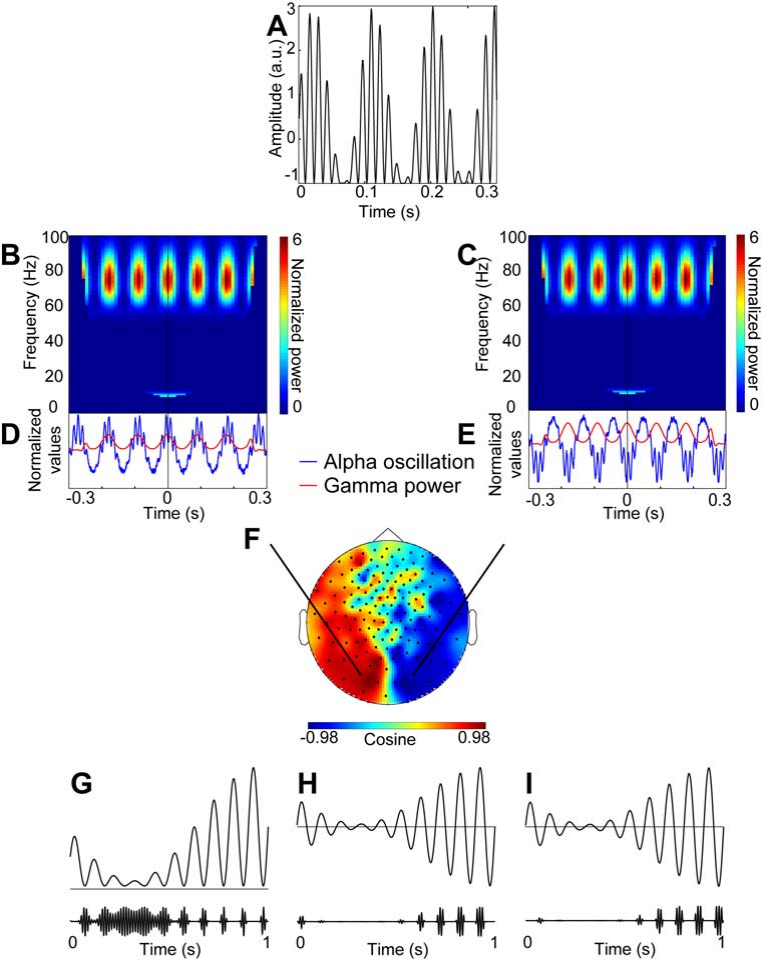
Simulation of the alpha/gamma interaction and hypothetical mechanisms explaining their interaction. A. A simulated signal in which gamma oscillations are modulated by the phase of an alpha signal. Using a forward model, the MEG signals were calculated with respect to a posterior dipole producing the coupled alpha-gamma signal. B,C. Time-frequency representations of power for epochs phase aligned with respect to the alpha cycles of the simulated signal. D,E. Time course of gamma power (red) overlaid with respect to the averaged epochs of the phase-aligned alpha oscillation (blue). Gamma power is the strongest at peaks of the alpha cycle to the left of the source (D). To the right of the source, the gamma power is the strongest at the alpha troughs (E). Gamma power has therefore the same phase relationship to the left and the right of the dipole whereas the polarity of the alpha signal is flipped. F. The topography of the phase difference (cosine of coherency) between alpha cycles and the time course of the gamma power originating from the source that generates the signal (A). The phase difference has a dipolar pattern similar to that observed in the measured MEG data (comparable to Fig. 2E). G. The first hypothesized mechanism for how alpha activity modulates gamma power in case when alpha peaks are modulated stronger than troughs. Gamma activity only occurs when the alpha signal is sufficiently low, e.g. at the troughs. The stronger the alpha the shorter the windows of gamma bursts. H. The second hypothesized mechanism for how alpha activity modulates gamma power when peaks and troughs of an alpha oscillation are modulated similarly. Gamma bursts occur at troughs of the alpha oscillation only when the alpha oscillation reaches a particular amplitude. I. The third hypothesized mechanism for how alpha activity modulates gamma power when peaks and troughs of an alpha oscillation are modulated similarly. Gamma bursts occur at peaks of the alpha oscillation only when the alpha oscillation reaches a particular amplitude.

To support the notion that the bipolar topography is a consequence of a single neuronal source whose gamma activity is modulated by the alpha rhythm we performed a simple simulation. We used a forward model (***G***) to calculate the fields (***F***) in the sensors given a dipolar source (***q***) at location **r**: ***F*** *=* ***Gq_r_*** *(t)*. For the forward model we used a spherical head model with a realistic position in the sensor array of CTF151 system. The source was located at the midline in ‘visual cortex’ (**r** = [−5,0,1] with respect to head coordinates) and pointing in the posterior direction. This forward model was applied to the signal
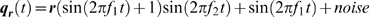
representing a gamma signal at *f_2_* = 75 Hz modulated by the phase of an alpha signal *f_1_* = 11 Hz (Fig. 3A). Notably, the resulting signal was asymmetric, i.e. alpha peaks were modulated differently than alpha troughs (see further). As for the measured data, the cross-frequency coupling was subsequently calculated for the sensor data. The topography of the cross-frequency coupling revealed a posterior distribution. The topography of the cosine of the coherency yielded a clear bipolar distribution (Fig 3F) which resembled that of the actual MEG data (Fig 2E). Further, similarly to the actual MEG data, gamma power on one side of the alpha source was modulated by the peaks and on the other side of the source by the troughs of the alpha oscillation (Fig. 3B,C,D,E). Even though the bipolar topography can be explained by a single dipole in the midline, it is an oversimplification since alpha activity emerges as a thalamocortical interplay [e.g., 21]. Nevertheless, MEG is mainly sensitive to neocortical and not thalamic sources. Furthermore, the midline alpha source is likely to be a consequence of summed activity from central sources in the right and left hemisphere.

**Figure 4:**
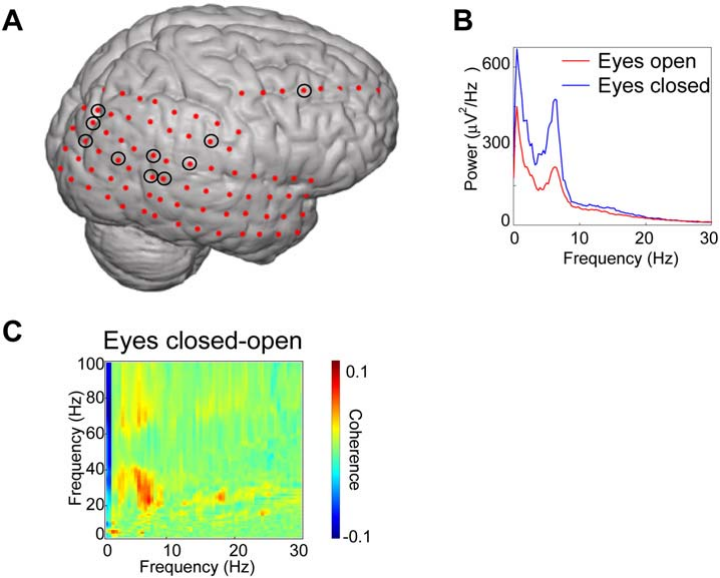
Cross-frequency coupling in intracranial data. A. The placement of the intracranial electrodes. The marked electrodes had at least a 10% increase in 5–10 Hz power when comparing eyes closed to open. B. The power spectra for the marked sensors (calculated using Welch methods; 50% overlapping Hanning window of 2048 points). Note the strong ∼7 Hz peak which increased with eyes closed. C. Cross-frequency analysis for eyes closed minus eyes open reveals that the phase of the ∼7 Hz rhythm modulates the 20–40 Hz power.

To support the MEG data we obtained a dataset from an epileptic patient with electrodes implanted intracranially. These electrodes covered a larger part of occipital and posterior parietal cortex (Fig 4A). To identify the equivalent of the alpha activity in the intracranial data we calculated the power spectra during eyes closed. We observed a strong peak ∼7 Hz in many of the electrodes. We then selected the electrodes in which the 5–10 Hz power increased more than 10% when comparing eyes closed to open (electrodes marked in Fig. 4A). The power spectra averaged over these electrodes are shown in Fig. 4B. We consider this activity the equivalent of the alpha activity, even though the frequency is lower due to the drugs and/or the pathology (see Discussion). We subjected the data from these electrodes to the cross-frequency analysis (Fig. 4C). The phase of the ∼7 Hz rhythms clearly modulated power at 20–40 Hz; this modulation was stronger for eyes closed compared to eyes open.

Further, to check the robustness of our method, we have investigated whether the settings used in the analysis may favor or hinder the detection of the alpha-gamma coupling in the MEG signal. Fig.S1 shows that cross-frequency coupling can be detected irrespectively of the number of cycles used to extract the envelope of the fast signal and the number of data points used to compute coherence.

## Discussion

Our findings show that gamma power in humans is phase-locked to ongoing alpha oscillations. By investigating the topography of the phase relationship of gamma power to alpha phase our findings can be explained by a single source in the posterior area. This source is producing a gamma signal which is modulated by the phase of the alpha rhythm.

To further support the findings we subjected intracranial data from an epileptic patient to the same analysis. Strong activity was seen around 7 Hz. We still consider this to be alpha activity due to the posterior distribution and the increase in power with eyes closed. Slowing of the alpha rhythm can be explained by the antiepileptic drugs and the pathology. For instance, the drug carbamazepine which was administered to the subject is known to reduce the alpha frequency [22], [23]. There are numerous cases in which slowing of alpha frequency is reported in epileptic patients [e.g. 24], [25], [26]. Based on these considerations, we will consider the 7 Hz activity in the patient the equivalent of the posterior alpha activity observed in healthy subjects. The cross-frequency analysis revealed a strong coupling between 20–40 Hz power and the phase of the 7 Hz activity. This 4–5 fold ratio corresponds to the ratio between 30–70 Hz gamma power and 8–12 Hz alpha phase observed in healthy subjects. We conclude, that strong alpha to gamma coupling can be observed in intracranial data albeit there is slowing in both the alpha and gamma band.

Alpha oscillations have been proposed to represent functional inhibition of the human visual system [reviewed in 27]. Note that functional inhibition reflects a reduction in neuronal processing which is not necessarily the same as GABAergic inhibition. Occipital gamma activity, in turn, although present in various tasks, particularly strongly manifests itself during visual processing [2], [28]–[30]. Although e.g, Chorlian et al. [31] have reported modulations in gamma power phase-locked to alpha oscillations during visual stimulation, our results demonstrate that these rhythms interact even in the absence of visual input. However, unlike in MEG studies on sustained visual attention [e.g.,18], no peak in the gamma band was observed in the power spectra: the gamma activity only became detectable when studied in relation to the alpha phase. What is the functional role of alpha modulating gamma power? As it has been proposed, e.g., by Klimesch et al. [e.g. 27], alpha activity might provide phasic inhibition of posterior areas. There are three possibilities (Fig 3 G,H,I):

1. Gamma burst responsible for visual processing can only occur when the alpha signal is low enough e.g. at the troughs (Fig. 3G). Thus, the periods of gamma activity become briefer with stronger alpha. When alpha is sufficiently weak, gamma can occur during the whole cycle. This, however, requires that the alpha signal is asymmetric (Fig. 3A), i.e. that peaks are modulated stronger than the troughs (or vice versa) [32], [33]. This notion is consistent with alpha activity reflecting functional inhibition. The bouts of gamma in each alpha cycle allow for some neuronal processing in posterior areas.
2. Alpha signal is symmetrically fluctuating around zero (peaks and troughs are modulated similarly) and gamma bursts occur at troughs only when alpha oscillation reaches a particular amplitude (Fig. 3H).
3. Alpha signal is symmetrically fluctuating around zero and gamma bursts occur at peaks only when alpha oscillation reaches a particular amplitude Fig. 3I).

It is physiologically plausible that spontaneous fast oscillations are produced by a network of neurons whose excitability is modulated by a slow rhythm. Such a mechanism is consistent with a computational model of White et al [34] in which concurrent fast and slow oscillations in the hippocampus are generated by neuronal circuits containing GABAergic interneurons with both fast and slow synaptic kinetics. Such network dynamics has been described in a number of brain structures. It is plausible that a related mechanism can be used to account for our findings.

In conclusion, our findings show that phase to power cross-frequency couplings can be observed in non-invasive as well as invasive recordings in humans. Further work investigating the interaction between alpha and gamma oscillations in cognitive tasks would help to elucidate the functional role of this phenomenon.

## Materials and Methods

### MEG data

Ongoing brain activity was recorded (sampling rate: 1200 Hz, low-pass filter: 300 Hz) using a whole-head MEG system with 151 axial gradiometers (VSM/CTF Systems, Port Coquitlam, Canada) from 14 young healthy volunteers (mean age 26.8±2.4, 8 females). In addition to the MEG, the electrooculogram (EOG) was recorded from the supra- and infra-orbital ridge of the left eye for the subsequent artifact rejection. The study was approved by the local ethics committee (Commissie Mensgebonden Onderzoek – Regio Arnhem Nijmegen, CMO 2001/095), and a written informed consent was obtained from the subjects according to the Declaration of Helsinki. Subjects were presented with tones (500 ms duration, 333 Hz). One tone indicated that a subject had to close his/her eyes; two tones indicated that the eyes should be opened. Only data from periods of eyes closed have been used in the subsequent analysis.

Partial artifact rejection was performed by rejecting segments of the trials containing eye-blinks, muscle and SQUID artifacts. By this procedure smaller segments, rather than a whole trial, can be rejected. In order to ensure that segments were sufficiently long for the subsequent analysis, segments shorter than 1 s were discarded as well. On average, 95.4 s±15 s of data underwent subsequent analysis. Independent component analysis (ICA) was used to remove heart artifacts and eye movements remaining after artifact rejection routines [35].

### Intracranial data

We obtained a data set from one subject (36 years old; female) who had been surgically implanted with subdural electrodes on the cortical surface. The patient had received the following drugs at the day of recording: Tegretol (carbamazepine) 1200 mg, Difantoine (phenytoine) 200 mg and Kefzol (antibioticum) 3000 mg. The electrodes were placed to best localize epileptogenic regions (see Fig. 4A). The iEEG signal was recorded with a 128 channel Micromed system with platinum electrodes (2.3 mm diameter) with an inter-electrode spacing of 1 cm. The signal was sampled at 512 Hz, and bandpass filtered between 0.15 and 134.4 Hz. The subject was asked to open and close the eyes in periods of 30 s respectively; this was repeated 5 times. Epochs with major epileptic artifacts were removed from the data and the electrodes over the subsequently resected areas were excluded from the analysis.

### Data analysis

In order to investigate cross-frequency interactions we have developed a tool calculating coherence between a low frequency signal and the time-course of the power at higher frequencies. Let the signal {*X_t_*} be represented by the time series *X_1_*, *X_2_*, …, *X_N_*. First, the time-course of power *S_1_(f_2_)*, *S_2_(f_2_)*, …, *S_N_(f_2_)* was estimated for frequency *f_2_* by applying a sliding tapered time-window followed by a Fourier transformation:
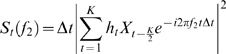



Here 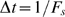 where 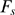 is the sampling frequency. The function 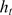 is a Hanning taper *K* data points-long equaling the length of the sliding time window. The length of the time-window decreased with frequency: 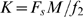 where *M* denoted the numbers of cycles per time window. We chose to use *M* = 7 cycles (for the intracranial data we used M = 6 cycles). Next, the coherency *Coh(f_1_,f_2_)* was estimated between signal {*X_t_*} and the estimate of the time-course of power{*S_t_(f_2_)*} for a given frequency *f_1_*. The coherency was calculated with respect to the two time series divided into *M* segments being *L* = 2048 data points long (for the intracranial data we used L = 1024 due to the lower sampling frequency):
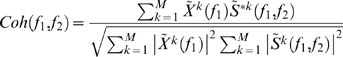
where
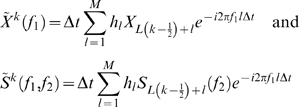



The coherence was the absolute value of the coherency 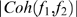. The phase difference between the signal at *f_1_* and the power at *f_2_* is given by the angle of the coherency 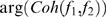. In this case 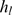 refer to a 2048 points Hanning window and ^*^ to the complex conjugate. This allowed us to characterize the phase-to-power cross-frequency interaction with respect to *f_1_* and *f_2_* sensor by sensor. To assess reliability of the estimated coherence, a statistical analysis was performed by randomly shifting the time course of signal {*X_t_*} (at least 3001 points) with respect to the estimated power {*S_t_(f_2_)*} and recalculating the coherence for the channel with the most pronounced effect (based on visual inspection). Repeating this 200 times yielded a distribution of coherence values. Further, a maximum coherence value between the alpha band and any of the frequencies between 2 and 100 Hz has been identified in every randomization. The proportion of the randomization coherence values above the coherence value to be tested corresponded to the p-value.

In order to assess phase relationship between alpha and gamma power, we bandpass-filtered the MEG data +/−4 Hz of the alpha peak frequency determined from the power spectra in each subject with acausal FFT filter. Next, 0.6 s of unfiltered epochs of data phase aligned to the alpha cycles were extracted. This was done by identifying the peaks of the alpha cycles in the bandpass-filtered data. Time-frequency representations (TFRs) of power were calculated for each segment using Fourier transforms calculated for short sliding time windows. Power estimates were averaged across trials. We applied a Hanning taper to an adaptive time window of 6 cycles for each frequency between 2–150 Hz (ΔT = 6/f). This analysis was done using the FieldTrip toolbox (http://www.ru.nl/neuroimaging/fieldtrip).

## Supporting Information

Figure S1 Cross-frequency plots for data from one representative subject calculated with different combinations of the number of cycles (4–7) that was used to extract the envelope of the fast signal, The number of data points (512–2048), was used to compute coherence between the envelope of the signal and the signal itself. Cross-frequency coupling can be detected reliably for a wide range of parameter settings.
(4.47 MB TIF)
