# Supplementary figures and images for: Gamma Power Is Phase-Locked to Posterior Alpha Activity
Source: PLoS One. 2008 Dec 22;3(12):e3990. doi: 10.1371/journal.pone.0003990 (PMC2602598; doi:10.1371/journal.pone.0003990)

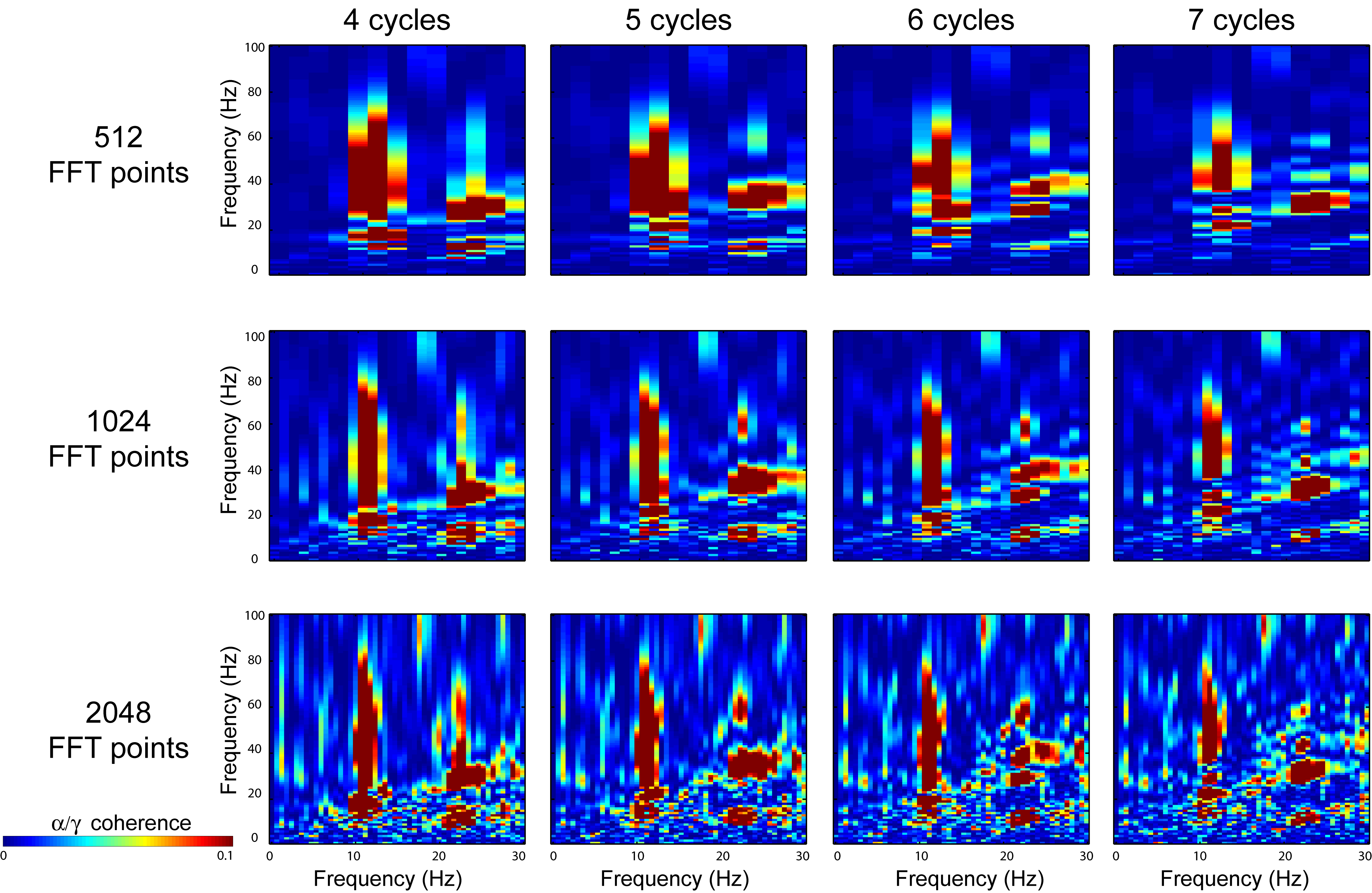

Supplement: Figure S1 — Cross-frequency plots for data from one representative subject calculated with different combinations of the number of cycles (4–7) that was used to extract the envelope of the fast signal, The number of data points (512–2048), was used to compute coherence between the envelope of the signal and the signal itself. Cross-frequency coupling can be detected reliably for a wide range of parameter settings. (4.47 MB TIF) [file pone.0003990.s001.tif]
